# Medicine

**Published:** 1909-12

**Authors:** George Parker


### Progress of the Medical Sciences.

### MEDICINE.

**Infectious or malignant endocarditis** of the chronic type is often peculiarly insidious in commencement, and may go on for months before a certain diagnosis can be made. Osler, to whom the recognition of these very prolonged cases is largely due, lays down[1] that there are two groups, one with irregular fever and "chills" of a malarial type, and the other with variable fever for four or even twelve months and often no "chills" at all. The disease is a true septicæmia, and the endocardial factor may be in the background. There may be a history of rheumatic fever, and in many an old valvular lesion; or there may be no symptoms for months except a slight daily rise of temperature, varied by occasional periods of apyrexia. Indeed, the patient may sometimes go about his work for a while feeling but little amiss. In ten cases reported by Osler there were only four where the diagnosis was confirmed by well-marked embolism, causing

[1] *Quart. J. Med.*, 1909, ii, 219.

an enlarged spleen, paralysis or hæmaturia, but seven showed transient erythematous nodules or wheals of a pink colour and with a white central point or area. These wheals are therefore an important indication when present. The disease may be mistaken for tuberculosis or typhoid, and blood examinations may be negative or show a strepto-, pneumo-, or staphylo-coccal infection, or even an influenzal or gonococcal one. All his cases ended fatally, but he recognises the possibility of successful vaccine treatment.

Horder[1] discusses the same subject in a report on 150 cases of infectious endocarditis. Besides ordinary acute attacks he notices a latent form where no symptoms during life may be found, a subacute group, and the true chronic form of six to eighteen months, duration or longer. In the subacute one there are usually gradual heart failure, prostration, rheumatic pains and pyrexia. In the chronic type we may have on the one hand loss of flesh, progressive anæmia, irregular fever, vague pains, red, tender patches of skin, and slight valve lesions; but on the other hand the general health may seem unaffected, and nothing conclusive may be found before some embolism occurs. An irregular, remittent fever is the usual type of pyrexia, which may run a very mild course and be absent for several days together. He considers that organisms in the blood can with care be detected in 90 per cent. of the patients, and draws attention to the frequent occurrence of the influenza bacillus and of streptococci of low virulence, viz. S. fæcalis, salivarius and anginosus. The presence of these organisms accounts for the absence of suppuration, the scanty leucocytosis and fever, and the chronic type of the disease. The prognosis is very bad in all forms. None of the ordinary remedies have any effect, and great hopes were formed that antisera would be efficacious, but so far they have failed, only one of Horder's cases having been cured, and in this one no definite organism had been isolated. As ordinary streptococcal sera are made from virulent streptococci, he suggests that better results may be hoped for from a serum derived from fæcal or salivary types and given by venous injections. Wright's vaccines have been tried, but Horder cannot report a single success in twelve cases, though Barr[2] claims one complete cure about which, indeed, serious doubts have been expressed. Horder has, however, seen considerable temporary improvement, and will in future give bactericidal sera at the same time as the vaccine.

Gilman Thompson[3] reports seven cases of infectious endocarditis treated by vaccines, and claims to have had a complete cure in three, with beneficial results in all. In the diagnosis he lays stress on the petechiæ, the marked anæmia, the harsh booming character of the heart murmur when present, and the

[1] *Ibid.*, p. 289. [2] *Lancet*, 1907, i, 499.
[3] *Am. J. M. Sc.*, 1909, cxxxviii, 169.

irregularity of the fever. His results are certainly extraordinary, and if similar success is obtained by other people a very different outlook will be given to the disease.

The instrumental measurement of **blood pressure** at the bed-side is altering our view of many diseases, and opening up a wide field for research. Without discussing the conditions under which the use of vasodilators is desirable and useful, it is well to know what is their exact effect on high blood pressure in the human body. It is quite useless giving them with the idea that a slight but lasting reduction can be brought about in the way that the alkalinity of a fluid can be gradually reduced by less or more acid added to it.

Edwin Matthew[1] has observed and reported the rapidity, the duration and the amount of reduction produced by fixed doses of the vaso-dilators in common use. Thus he finds that two minims of liquor trinitrini commence on the average to act in one minute, and produce a maximum fall in four and a half minutes, which usually amounts to about 28 mm., and at once begins to pass off, so that the original level is reached in thirty minutes. With two or three grains of sodium nitrite, the fall commences in five minutes, reaches a maximum in fourteen, when it measures 32 mm. This, however, continues for about forty-five minutes, and passes off entirely in two hours. Half a grain of erythrol begins to act in five and a half minutes, and the maximum fall is reached in twenty-two, when it amounts to 35 mm., which persists for an hour or two. The original level is not reached till six hours have elapsed.

He reminds us of a well-known fact that in certain conditions little or no dilator action is produced by these drugs, and harm results if excessive doses are used to force a result. An instance of this may be seen in some advanced cases of granular kidney, and occasionally in advanced arterio-sclerosis, though not in all, as W. E. Russell notices. Matthew found that no effect followed the administration of tablets of certain good brands, apparently because they were hard and insoluble ; again, as long as much œdema existed very little effect was produced. He claims that in suitable cases sodium nitrite or erythrol in the above doses, given three times a day, will cause a reduction of pressure throughout the twenty-four hours. It is not clear how this results, as neither of them has an eight-hour period. On the whole, he found that a fall of about 30 mm. was usually sufficient to relieve headaches, giddiness and other troubles, and that if maintained the patient's general condition improved. Hence the above doses were chosen.

His experiments do not show that any permanent reduction results from prolonged administration, such as has been claimed for guipsine, an extract of mistletoe. Trinitrine indeed soon loses

[1] *Quart. J. Med.*, 1909, ii, 261.

its effect except in increased doses. Perhaps the most important question is whether a short daily relief of pressure by these drugs strengthens the vessels and saves or puts off a permanent decay of the arterial system apart from their temporary value in emergencies. The improvement seen under the use of regular purgatives may be an action of this kind, or it may be due to increased elimination of toxins, though the latter view seems insufficient to account for the results. There are some cases where the rise of pressure is compensatory, as perhaps in early Bright's disease, but there are others where it is nothing of the kind. In the latter there is good reason for combating the high tension.

**The treatment of diabetes** is the subject of an interesting paper by Janeway.[1] He aims at estimating the severity of both the disturbances of metabolism present, viz. the degree of glycosuria and that of acidosis. Thus he recommends all patients to be put first on a test diet of proteid and plenty of fat, equal to about thirty-five calories per kilo of their weights, and to this is added three ounces of white bread. (1) If the sugar disappears on this diet the glycosuria is of the mild type and acidosis is negligible. For these patients bread is cautiously increased till we find the amount they can take without producing sugar. Next we should test which variety of starch, *e.g.* potato, rice, oatmeal, the individual best tolerates, the ideal being to prevent glycosuria for long periods altogether. Occasional periods of strict diet should be enforced, but meanwhile we must maintain the patient's nutrition. If the sugar does not vanish with the low diet we may require to cut off even the three ounces of white bread, and in (2) *moderately severe* cases this will suffice. In (3) *really severe* ones we must proceed to reduce the proteid. There will be needed in (2) 2,000 calories of fat, or seven ounces, for an average man, and not more than 600 of proteid, or five ounces. This can be managed on a carefully planned dietary, but the fat, whether butter, bacon, cream or oil, often disagrees, and may require some alcohol to aid its digestion. Every effort must be made to get the patient's intelligent co-operation. It will be necessary in (3) to reduce the proteid given above for a time, say to three ounces or less, and in such cases occasional days of very low diet are most useful. On these nothing but eggs, bacon, greens, coffee and alcohol should be given.

As to the acidosis, a daily watch must be kept by the ordinary tests. It is claimed that the amount of the abnormal acids can be very simply and easily obtained by Folin's method for estimating ammonia. A still simpler method is described by Mathison in the *British Medical Journal*, 1909, vol. i, p. 715. If then the ammonia amounts to two, three or more grammes a day, or a marked ferric chloride reaction is present, strict diet periods must be shortened, and sudden changes in

[1] *Am. J. M. Sc.*, 1909, cxxxvii, 313.

carbohydrates avoided. Bicarbonate of soda half to one ounce daily should be given, proteid restricted and occasional days of low diet employed. The butter, too, should be thoroughly washed to get rid of any butyric acid; but to allow a full carbohydrate diet almost always fails to do good.

In every type of the disease we should endeavour to reduce the sugar to a minimum. Diabetic breads are of very little value, and can be replaced by smaller amounts of white bread.

Rudisch[1] thinks that a strict diet should be employed, but would reduce the amount of calories, especially of fat, finding that diabetics actually do better with a very spare diet. Tomato and other vegetable soups, and in some cases vegetable fats, are very useful. He claims that under daily doses of atropine, sugar disappears much more rapidly than with a carbohydrate free diet alone, if it is given cautiously and in increasing amounts up to the limit of tolerance. Jacobi confirms his results, and gives the tincture of belladonna to children till the cheeks flush after each dose without dilatation of the pupils. Both writers speak of the value of bicarbonate of soda in all cases, and of opium in many.

GEORGE PARKER.

### SURGERY.

[1] *J. Am. M. Ass.*, 1909, liii, 1366.

